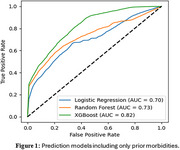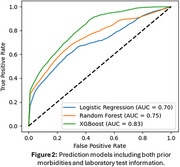# Predicting Future Risk of Dementia Using Machine Learning and Longitudinal Electronic Health Records

**DOI:** 10.1002/alz70856_103360

**Published:** 2025-12-26

**Authors:** Zehao Ye, Amelia Zai, Biqi Wang, Honghuang Lin

**Affiliations:** ^1^ University of Massachusetts Medical School, Worcester, MA, USA

## Abstract

**Background:**

Early diagnosis of dementia is crucial for facilitating timely interventions, potentially delaying progression, and improving patient outcomes. Electronic health records (EHRs) offer comprehensive, longitudinal data on patient health, which can be instrumental in identifying factors associated with the risk of developing dementia. This project aims to utilize machine learning techniques and longitudinal EHR data from a large hospital system to predict the risk of developing dementia.

**Method:**

We utilized EHR data collected from UMass Memorial Medical Center between January 1, 2017, and December 31, 2024. Patients diagnosed with dementia were identified based on ICD‐10 codes. We excluded patients younger than 65 years old from the analysis. For each dementia case, we selected ten referent patients matched by age, sex, and race/ethnicity. Patients without at least one year of health records prior to the dementia diagnosis or the last follow‐up (whichever came first) were excluded from the analysis.

Three machine learning algorithms are employed to build predictive models for the future risk of dementia, including logistic regression, random forest, and XGBoost. A total of 21 features were incorporated, including prior morbidities (e.g., diagnoses of hypertension, diabetes, and mental disorders) and laboratory test information (e.g., fasting glucose, low‐density lipoprotein, and triglycerides). Model performance was assessed using 5‐fold cross‐validation.

**Result:**

This study included 30,162 participants (mean age 80 ± 12 years old, 69.7% women, 9.4% of non‐white). Three models are evaluated to predict dementia risks. Figure 1 illustrates predictive performance with prior morbidities, where XGBoost achieved the highest performance with an area under the curve (AUC) of 0.82. Figure 2 demonstrates the predictive performance after incorporating lab test information, resulting in improved performance across most of the models. XGBoost again delivered the best results with an AUC of 0.83.

**Conclusion:**

We developed multiple machine learning models to predict the risk of dementia using EHR data from a large hospital system. Our findings highlight the potential of EHRs for early dementia diagnosis. These results should be validated further in additional hospital systems.